# A Comparative Study between Transcutaneous Electrical Nerve Stimulation and Fentanyl to Relieve Shoulder Pain during Laparoscopic Gynecologic Surgery under Spinal Anesthesia: A Randomized Clinical Trail

**DOI:** 10.1155/2018/9715142

**Published:** 2018-03-18

**Authors:** Zahra Asgari, Zahra Tavoli, Reihaneh Hosseini, Masoomeh Nataj, Fatemeh Tabatabaei, Fatemeh Dehghanizadeh, Hosein Haji-Amoo-Assar, Mahdi Sepidarkish, Ali Montazeri

**Affiliations:** ^1^Department of Obstetrics and Gynecology, School of Medicine, Tehran University of Medical Sciences, Tehran, Iran; ^2^Department of Anesthesiology, Tehran University of Medical Sciences, Tehran, Iran; ^3^Department of Obstetrics and Gynecology, Tabriz University of Medical Sciences, Tabriz, Iran; ^4^Department of Physical Medicine, Tehran University of Medical Sciences, Tehran, Iran; ^5^Department of Physical Medicine, Lorestan University of Medical Sciences, Lorestan, Iran; ^6^Department of Epidemiology and Reproductive Health, Reproductive Epidemiology Research Centre, Royan Institute for Reproductive Biomedicine, ACECR, Tehran, Iran; ^7^Population Health Research Group, Health Metrics Research Centre, Iranian Institute for Health Sciences Research, ACECR, Tehran, Iran

## Abstract

**Background:**

Traditionally, laparoscopic procedures have been performed under general anesthesia. Spinal anesthesia is an effective alternative to general anesthesia. However, one of the intraoperative complications of performing laparoscopic surgery under spinal anesthesia is shoulder pain. This study aimed to compare the effect of transcutaneous electrical nerve stimulation (TENS) with fentanyl on pain relief in patients who underwent gynecologic laparoscopy under spinal anesthesia.

**Methods:**

We conducted a prospective randomized clinical trial from May 2016 to March 2017. A sample of patients who underwent gynecological laparoscopy under spinal anesthesia was recruited. If they had shoulder pain, they randomly received either transcutaneous electrical nerve stimulation (TENS) or 50 mg of fentanyl. Pain intensity was measured using the single item visual analogue scale (VAS-10 cm) immediately before and 5, 10, 20, and 30 minutes after treatment. Also, the effect of higher doses of analgesia on pain relief was analyzed.

**Results:**

In all, 80 patients (40 patients in each group) were entered into the study. The mean pain intensity score was 9.02 ± 1.32 in the TENS group and 8.95 ± 1.33 in the fentanyl group at baseline (*P* = 0.80). Repeated measures analysis of variance indicated that there was no significant difference on overall pain scores between the two treatment groups adjusted for age, BMI, total analgesia used, and baseline pain score (*F* (1, 74) = 1.44, *P* = 0.23). The use of analgesic drugs in the TENS group was significantly higher than the fentanyl group (*P* = 0.01). In addition, we found that nine patients (22.5%) complained of nausea/vomiting in the TENS group compared to thirteen patients (32.5%) in the fentanyl group (*P* = 0.31).

**Conclusions:**

The findings indicated that TENS was not superior to fentanyl for pain relief in laparoscopic surgery. It seems that the correct use of TENS parameters might merit further investigation. This trial is registered with: IRCT2016031216765N3.

## 1. Background

Although general anesthesia is a choice for laparoscopic surgeries, regional anesthesia provides more benefits over general anesthesia [1, 2]. Spinal anesthesia is one of the common forms of regional anesthesia to perform lower abdominal surgeries. Benefits of spinal anesthesia include lower cost, prevention of airway manipulation, early ambulation, conscious and awake state of patients, lower postoperative analgesia, minimal nausea and vomiting, intact respiratory control mechanism that is very economical, and easy administration [3]. Despite these benefits, patient's discomfort due to shoulder tip pain is a limiting factor for use of spinal anesthesia in laparoscopy [4]. The prevalence of shoulder pain is reported to vary from 35 to 80%, and it remains up to 72 hours after surgery [5].

Different procedures have been evaluated in several studies to relieve shoulder tip pain in laparoscopy under spinal anesthesia [6]. A number of studies focused on surgical techniques, pressure, temperature and humidity of pneumoperitoneum, and drainage and different maneuvers to reduce the phrenic nerve stimulation [7–10]. Other studies evaluated the administration of drugs to prevent shoulder pain [11–16]. Several studies examined the effect of different drugs, acupuncture, and physiotherapy on postoperative shoulder pain. [11, 12, 17–19]. However, studies on the treatment of shoulder pain during laparoscopy are limited.

Currently, there are several treatment procedures for relieving acute pain during gynecologic laparoscopy. These include nerve blocking, drug therapy (narcotic medication such as fentanyl), and bioelectric therapy (transcutaneous electrical nerve stimulation-TENS). Of these, nerve blocking is an invasive approach and is difficult to perform; using narcotics is associated with some complications. Yet, TENS is a safe and effective technique and could reduce postoperative analgesic consumption [9, 20, 21]. Although a few studies are available on the benefits of TENS in relieving postoperative pain [22–24], at present we could not identify any studies on the benefits of TENS in relieving shoulder tip pain during laparoscopy. Therefore, we decided to evaluate the effectiveness of TENS on shoulder pain relief during gynecologic laparoscopy as compared to a narcotic therapy using fentanyl.

## 2. Methods

### 2.1. Trial Design

A prospective open parallel group randomized clinical trial was conducted at a teaching hospital, affiliated to Tehran University of Medical Sciences from May 19, 2016 to March 19, 2017. The study was approved by Ethics Committee of Tehran University of Medical Science (ID: 1394.2139) and was registered at Iranian Registry of Clinical Trials (IRCT2016031216765N3).

### 2.2. Participants

Women aged 18 to 50 years old who were candidate for simple gynecologic laparoscopy were entered into the study. Patients were excluded if they had neurological disease, spinal deformity or history of operation on the spine, body mass index above 35, pregnancy, history of allergy, or any contraindication to spinal anesthesia (coagulopathy and infection). Written informed consent was obtained from all participants.

### 2.3. Procedure

The surgical procedures included gynecological laparoscopy under spinal anesthesia. Spinal anesthesia was performed with a 25G spinal needle and after free flow of cerebrospinal space fluid. 4 ml (20 mg) of bupivacaine 0.5% was injected into L2-L3/L3-L4 subarachnoid at a rate of 0.1 ml/s. We operated all patients with pneumoperitoneal pressure up to 10 mmHg. In preoperative visit, the anesthesiologist explained the patients that any pain or discomfort occurring during surgery would be treated with intravenous medications or TENS. Patients were asked to inform the nurse anesthetist when they experience pain. The extra analgesic drug was 50 mg fentanyl and was administered intravenously.

### 2.4. Interventions

In the TENS group, 4 pads of the device with size of 4 × 10 cm and at the distance of 5 cm are adhered in the zone of scapula and behind both shoulders. TENS parameters were pulse frequency of 16 Hz and pulse width of 150 microseconds (1 burst per second, 8 pulses per burst). After spinal anesthesia, TENS device was turned on for 20 minutes for patients who complained of pain. Pulse amplitude was increased and adjusted according to patient's tolerability by the nurse (the highest level that did not make patients uncomfortable). The electrical impulse was applied up to 20 minutes for patients who complained of pain.

In the fentanyl group, 50 milligrams (mg) of intravenous fentanyl was injected.

TENS or fentanyl were administered during surgical procedures when patients experienced unbearable pain and requested pain relief.

### 2.5. Outcome Measure

The primary outcome of the trial was shoulder pain relief. Pain was measures using a visual analogue scale (VAS). The pain VAS is a continuous scale comprised of a horizontal (HVAS) or vertical (VVAS) line, usually 10 centimeters (100 mm) in length, anchored by 2 verbal descriptors, one for each symptom extreme [25]. A nurse recorded the VAS scores after patients indicating pain severity by their fingers at baseline and 5, 10, 20, and 30 minutes after interventions. The nurse who recorded the VAS scores (and the other outcomes) was blinded to group allocation.

The secondary outcome was amount of extra analgesic use and nausea and vomiting. The former was measured on the basis of total usage of fentanyl (mg) while the latter was indicated by the patients.

### 2.6. Randomization

Patients were randomized in 1 : 1 ratio to receive TENS or fentanyl. Allocation sequence was done using a computer-generated randomization chart by the coordinating investigator of research center at hospital. Permuted block randomization with a block size of six was used.

### 2.7. Sample Size

Sample size was estimated according to the VAS score as a primary outcome. A repeated measures design with one between-subjects factor (intervention groups) and one within-subjects factor (times of recording) needed 40 patients per each intervention groups (a total sample of 80 patients) in order to achieve 80% of power to detect average differences of 1 score in VAS in two treatment groups (an effect size of 0.23). The standard deviation of the VAS score in each treatment groups was assumed to be 4 and equal in both treatment groups. All above calculation assumed significance level of 0.05.

### 2.8. Statistical Analysis

The analysis was by intention-to-treat and included all patients who were randomly assigned. Categorical and continuous variables were summarized as proportions and mean ± standard deviation, respectively. Student's *t*-test and chi-square were applied to compare baseline characteristics and side effects among groups. The primary outcomes (mean values of VAS scores) were analyzed using repeated measures analysis of variance. The model included treatment as fixed factor and age, BMI, analgesia usage, and baseline VAS score as covariates. All statistical analysis was performed using Statistical Package for the Social Sciences (SPSS; SPSS Inc., USA) version 18, and *P* value of <0.05 was considered statistically significant.

## 3. Results

**Figure 1:**
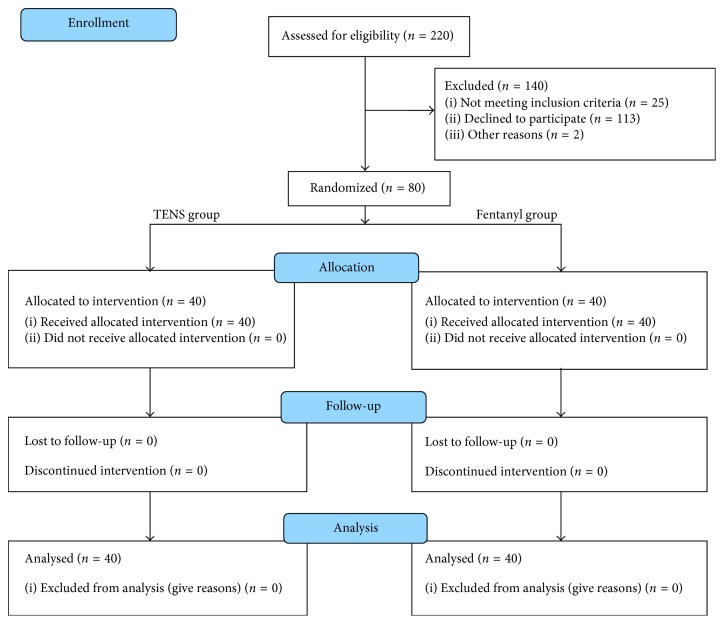
Flowchart of the study.

Figure 1 shows the trial profile. In all, 80 patients were entered into the study. Of these, 40 patients were assigned to the TENS group, and 40 patients included in the fentanyl group. Both groups were well matched with respect to age, BMI, surgery duration, and type of operation (Table 1).

At baseline, the mean pain intensity level was 9.02 (SD = 1.32) in the TENS group while in the fentanyl group it was 8.95 (SD = 1.33) indicating no significant differences between the two groups (*P* = 0.80). However, a significant difference was observed at 5-minute evaluation indicating that fentanyl was more effective in reducing pain severity (*P* < 0.001) while at 30-minute assessment the TENS showed a significant superiority (*P* = 0.008). The results are shown in Table 2.

Further analysis showed that the overall analgesia usage was significantly higher in the TENS group than the fentanyl group (51.2 mg in TENS group versus 33.7 mg in fentanyl group, *P* = 0.013). In addition, in the TENS group, nine patients (22.5%) complained of nausea/vomiting compared to thirteen patients (32.5%) in the fentanyl group (*P* = 0.31).

Finally, comparing pain severity in different assessment points by performing repeated measures analysis of variance indicated no significant differences on overall VAS scores between treatment groups adjusted for age, analgesia usage, BMI, and baseline pain score (*F* (1, 74) = 1.44, *P* = 0.23).

## 4. Discussion

Shoulder pain is one of the intraoperative consequences of spinal anesthesia in laparoscopic surgeries [26, 27]. The etiology of such pain is thought to be subdiaphragmatic irritation of the peritoneum by CO_2_ and stretching or injury [8, 27]. The present randomized clinical trial evaluated the efficacy of transcutaneous electrical nerve stimulation (TENS) to decrease intraoperative shoulder pain compared to fentanyl. TENS is a noninvasive, safe, and inexpensive analgesic technique for treatment of acute and chronic pain [28], and fentanyl is a narcotic used for the analgesic treatment of shoulder pain through local anesthesia combined with IV injection [29].

Unfortunately, we could not identify any study comparing TENS and fentanyl for shoulder pain relief in patients undergoing spinal anesthesia. Previous investigations using TENS usually focused on postoperative pain, and studies using fentanyl generally focused on prevention of shoulder pain. Perhaps, the current investigation is the first study that compares TENS with fentanyl as analgesia to relieve shoulder pain in laparoscopic procedures.

Overall previous investigations using TENS for pain relief could be categorized in three groups: using TENS for pain relief comparing with placebo, comparing TENS with no treatment, and comparing TENS with pharmacological drugs. Two trials examined the effect of TENS compared with placebo and identified significant postoperative pain relief in active TENS group. In addition, the consumption of analgesics in poststimulation time was greater in inactive TENS group [30, 31]. Five trials showed that TENS resulted in greater pain relief compared to no treatment [32–36]. De Angelis et al. [33] showed reduction in pain intensity during hysteroscopy under TENS than a group without treatment. Amer-Cuenca found that TENS was effective over 50% on pain relief associated with colonoscopy compared to a group without treatment. In contrast, two trials found no significant differences between TENS and a group without treatment on pain intensity during flexible cystoscopy and during screening flexible sigmoidoscopy [37, 38]. Three out of four studies reported an improvement, at least one outcome measure in favor of TENS when comparing it with a nonopioid pharmacological treatment [35, 39–41]. To this end, one might conclude that TENS was effective in pain relief compared to placebo, no treatment, and nonopioid pharmacological treatment. However, to give an impression of studies using fentanyl for shoulder pain a brief description is discussed.

Studies have shown that trials that used fentanyl for prevention of shoulder pain was not effective in all patients and in some cases additional dose of fentanyl were required. The study by Van Zundert et al. on 20 patients under spinal anesthesia and fentanyl (50–100 mg) reported complains of shoulder pain in five patients [29]. In addition, Lee et al. showed that in 11 patients with thoracic epidural analgesia, six patients required 50 mg of fentanyl, five patients required additional dose of fentanyl, and one patient required conversion to general anesthesia because of severe shoulder pain [26]. According to Tzovaras et al., out of 50 patients under lumbar spinal anesthesia with opioids, 43% required additional dose of fentanyl [42]. However, in the present study, we found that 57.5% of patients in fentanyl group needed excessive dose of opium.

Using more opioids might be both costly and harmful to patients. Thus, the current randomized trial was an attempt to either reduce the use of fentanyl or if possible replace it with TENS, although we could not show that TENS was superior to fentanyl. Several explanations could put forward to justify the results. First, we studied intraoperative pain and stimulator of pain continued up to the end of the study. Thus, TENS was not effective as expected. Secondly, it could be due to the fact that the maximum effect of TENS possibly can be obtained in long term while patients usually could not tolerate the pain and therefore ask for more analgesia. Thirdly, we compared TENS with a very powerful analgesic drug (fentanyl) and thus could not see any superiority for the TENS group. Finally, as we know, the mechanism of pain relief produced by TENS is activation of the endogenous opioid pathway, and this might be another reason for a lesser effect when compared to fentanyl. However, to see the exact effect of TENS, we propose a different study design. Perhaps a study with a group of patients receiving TENS plus narcotics and another group receiving narcotics only might indicate whether TENS could result in reduction of opium consumption while giving similar or better pain relief during laparoscopic surgery. Another suggestion is to reapply TENS more than one session.

We used low-frequency electrical stimulation in order to avoid muscle contraction and to make it bearable for the patients. Such approach for using the TENS could be problematic since one might argue that the effect (benefits) of TENS depends on the correct use of its parameters [43].

The findings indicated that patients in both groups experienced nausea and vomiting as expected [44, 45]. However, the number of patients who experienced nausea and vomiting in the fenanyl group was higher than the TENS group. Such observation, although not significant, might be attributed to the excess usage of narcotic drug by the fentanyl group as compared to the TENS group. In fact, the fentanyl group received a 50 mg fentanyl as intervention and 33.7 mg as excessive analgesia to overcome pain during surgery.

## 5. Study Limitation

During administration of TENS, although pulse amplitude was increased and adjusted according to patient's tolerability, we did not increase the intensity during the treatment every time, and thus this might make pain relief effect limited. This should be seen as a limitation of the study in addition to the short time of evaluation of the pain.

## 6. Conclusion

The findings suggest that TENS was not superior to fentanyl. Further evaluation with a larger number of patients, using correct parameters of TENS, and more focus on opioid dosage is recommended.

## Figures and Tables

**Table 1:** Patients' characteristics.

	TENS (*n* = 40)	Fentanyl (*n* = 40)
Age, mean (SD) (years)	31.35 (4.89)	31.15 (6.28)
Body mass index, mean (SD) (kg/m^2^)	26.23 (4.18)	26.11 (4.37)
Surgery duration, mean (SD) (minutes)	51.62 (11.89)	45.82 (19.78)
*Type of operation*, no. (%)		
Ectopic pregnancy	21 (52.5)	22 (55)
Infertility	7 (17.5)	10 (25)
Ovarian cysts	6 (15)	1 (2.5)
Ovarian torsion	6 (15)	7 (17.5)

**Table 2:** Comparing pain score in TENS and fentanyl groups as measured by the visual analogue scale (VAS)^∗^.

	TENS (*n* = 40)	Fentanyl (*n* = 40)		
	Mean (SD)	Mean (SD)	95% CI of the difference	*P*
Pretreatment Pain	9.02 (1.32)	8.95 (1.33)	−0.51 to 0.66	0.80
Posttreatment pain (5 minutes)	7.62 (2.33)	5.52 (2.18)	1.09 to 3.10	<0.001
Posttreatment pain (10 minutes)	3.92 (2.53)	3.55 (2.18)	−0.67 to 1.42	0.48
Posttreatment pain (15 minutes)	1.72 (2.11)	2.60 (2.35)	−1.86 to 0.11	0.08
Posttreatment pain (30 minutes)	0.65 (0.89)	1.50 (1.75)	−1.46 to −0.23	0.008

^∗^Higher values indicate higher pain severity.

## Data Availability

The data is available from corresponding author in request.
